# Opinion on “Artificial intelligence in sepsis early prediction and diagnosis using unstructured data in healthcare”

**DOI:** 10.3389/fimmu.2025.1629766

**Published:** 2025-09-15

**Authors:** Aifeng He, Leiming Xu, Shengkai Yang

**Affiliations:** ^1^ Department of Emergency, Affiliated Binhai Hospital, Kangda College of Nanjing Medical University, Yancheng, Jiangsu, China; ^2^ Department of Neurosurgery, Affiliated Binhai Hospital, Kangda College of Nanjing Medical University, Yancheng, Jiangsu, China

**Keywords:** artificial intelligence, sepsis, early prediction, diagnosis, sepsis early risk assessment

## 1 Ethical and operational implications

The authors note a 17% reduction in false positives, yet the clinical consequences of residual false alarms, a persistent challenge in AI deployment, warrant further analysis. False positives can increase clinician cognitive load, contribute to alarm fatigue, and potentially erode patient trust when interventions are triggered unnecessarily. For example, AI models may misclassify non-infectious systemic inflammatory response syndrome (SIRS) as early sepsis, prompting unnecessary antibiotic administration or invasive monitoring, thereby straining resources and exposing patients to avoidable risks (2, 3). In intensive care units (ICUs), they may also lead to inefficient resource allocation, including unwarranted antibiotic use or invasive monitoring. Existing frameworks such as the Ethics Guidelines for Trustworthy AI (European Commission, 2019) (4) or The AMA’s Policy on Augmented Intelligence in Health Care (2019) (5) provide valuable reference points. These emphasize transparency, accountability, and shared decision-making, which could be embedded into AI deployment strategies to mitigate these risks.

## 2 Interpretability of LDA-derived topics

The application of LDA for topic modeling in unstructured notes is innovative; however, explainability remains a key barrier to clinical adoption. While the authors use LDA to extract latent topics from clinical narratives, the study lacks explicit detail on whether these topics were validated or manually labeled to confirm alignment with known clinical indicators of sepsis. This is a critical gap, as the interpretability of extracted topics determines clinician trust and model transparency. For instance, clinical variables such as rising heart rate (tachycardia), respiratory rate (tachypnea), altered mental status, or low blood pressure are often early signs of sepsis. If an AI model highlights these parameters—or their semantic equivalents in unstructured notes—as predictive features, clinicians are more likely to trust and act on the output.

Conversely, if the model relies on opaque or non-clinical latent topics, adoption may be hindered (6). For example, topics dominated by administrative language such as “insurance documentation”, “bed transfer”, or “discharge planning” could appear predictive due to correlations in the training dataset, yet lack direct physiological relevance to sepsis (7). If such opaque topics were emphasized without clinician oversight, they could undermine trust, leading physicians to discount the algorithm’s recommendations (8–11). Enhancing transparency through topic coherence scores, clinical expert annotation, and mapping extracted features to established ontologies like SNOMED-CT would help translate complex AI decisions into actionable insights. Future work should prioritize *post hoc* labeling of LDA topics and associate them explicitly with sepsis-relevant pathophysiological constructs to bridge the gap between machine reasoning and clinical intuition.

## 3 External validation in diverse healthcare settings is essential

The study’s single-center design limits generalizability, as variations in EMR systems, documentation practices, and patient populations across institutions could affect model performance. External validation in diverse healthcare settings is essential. Additionally, while the algorithm’s accuracy is impressive, its “black-box” nature may hinder clinical adoption. Explainability, such as identifying which topics or variables drive predictions, could bridge this gap. Ethical and operational challenges, such as false positive management and liability concerns, also warrant attention. For instance, while the algorithm reduces false positives by 17%, their impact on resource utilization remains unexplored. Finally, comparative benchmarking against other AI sepsis tools, such as Sun B et al.’s prediction of sepsis model (12), would clarify SERA’s unique contributions.

Future research should build on this work by exploring multimodal data integration, like real-time vitals and wearable devices, to further refine predictions, particularly in the critical 4–6 hours window before sepsis onset (13, 14). To support cross-institutional validation and protect patient privacy, federated learning frameworks, such as FedAvg or SplitNN, can be employed to train decentralized models across institutions without transferring raw patient data. Dynamic risk stratification, where predictions update in real-time with new clinical data, could enhance responsiveness. Additionally, adapting the model for low-resource settings, where sepsis burden is high but EMR infrastructure is limited, would broaden its global impact (15), lightweight NLP models such as DistilBERT, MobileBERT, or TinyBERT can be adapted for local deployment, offering efficient language processing with reduced computational overhead (16–18). These models can extract sepsis-relevant clinical patterns from brief physician notes or basic triage descriptions. Additionally, real-time streaming pipelines using platforms like Apache Kafka, Apache Flink, or TensorFlow Serving can facilitate continuous data ingestion and model updates, enabling near-instantaneous risk recalibration. By adopting these scalable, efficient strategies, future iterations of the SERA algorithm may achieve broader utility across diverse healthcare ecosystems, including resource-limited settings and distributed hospital networks.

## 4 Causal relevance and target trial emulation

While the SERA algorithm demonstrates strong predictive performance, whether early identification of sepsis causally improves clinical outcomes remains an open question. Most existing models, including SERA, are evaluated using retrospective association metrics such as AUC, sensitivity, and specificity, which do not guarantee that earlier prediction will lead to better patient outcomes. To address this, emerging frameworks such as target trial emulation offer a promising approach to infer causal relationships from observational data. Specifically, retrospective ICU datasets containing timestamped interventions such as antibiotic initiation, fluid resuscitation, or admission to the ICU could be used to emulate randomized controlled trials (19–21). Patients with comparable baseline risk profiles could be contrasted based on whether they received earlier intervention following AI-based alerts versus standard care (22). This methodology simulates randomized controlled trials using routinely collected clinical data, providing more robust evidence of effectiveness (23). Moreover, causal inference methods such as inverse probability weighting (24, 25), g-computation (26), and marginal structural models (27) may further support estimation of the effect of early prediction on sepsis-related morbidity and mortality. Applying these tools to intervention-timestamped ICU data would allow researchers to evaluate whether timely alerts from the SERA algorithm result in earlier antibiotic administration, fluid management, or escalation of care, and ultimately improve survival and reduce complications (1, 28–30). Future research incorporating these methods is essential to bridge the gap between prediction and clinical impact.

## 5 Conclusion

Goh et al. have developed a sophisticated AI tool that addresses a critical unmet need in sepsis care. Their work highlights the untapped potential of unstructured clinical data and sets a new benchmark for early sepsis prediction. Nevertheless, the reliability and equity of deployment will hinge on explicit management of training-data quality, documentation heterogeneity, and clinical bias—with performance and calibration reported across pediatric, geriatric, and non-English documentation cohorts. Coupling these safeguards with rigorous external validation, enhanced model interpretability, and deeper consideration of ethical and operational challenges. By incorporating domain-specific explainability, ethical safeguards, and practical deployment strategies, along with causal validation frameworks such as target trial emulation, future research can transform SERA into a scalable, dynamic, and globally applicable AI solutions for sepsis and other time-sensitive conditions.
